# PB.17. Are patients who have had total body irradiation at similar risk of breast cancer to those having mantle radiotherapy? A review of the evidence and suggestions on breast imaging surveillance

**DOI:** 10.1186/bcr3736

**Published:** 2014-11-03

**Authors:** J Muscat, G Rubin

**Affiliations:** 1 Brighton and Sussex University Hospitals NHS Trust, Brighton, UK; 2 Mater Dei Hospital, Msida, Malta

## Introduction

There are an increasing number of total body irradiation (TBI) survivors. UK haematology and oncology units follow international guidelines and advise that they should have annual mammography from age 40.

## Methods

A literature review found a single large study published in *Blood *in 2008 based on 3,337 women from 83 centres, who had survived at least 5 years after transplant [1]. The radiation dose was 8 to 16 Grey distributed homogeneously rather than to the mediastinum in mantle radiotherapy. Fifty-two women developed breast cancer at a median of 12.5 years after transplantation, and 47 of these had TBI as well as bone marrow transplant. Their breast cancer incidence was compared with the standardised incidence.

## Results

The study found that: overall breast cancer risk was moderately increased, by 2.2 (95% CI = 1.7 to 2.9); risk was concentrated in younger patients, those treated in their teens and twenties; and the standardised incidence of those presenting after 20 years was also 10-fold that expected.

## Conclusion

There are similarities between the risks of TBI and mantle radiotherapy, cancers occurring if irradiated when young and cumulative risk increasing disproportionately with length of follow up. More work needs to be done comparing the radiation dose to the breast and relative breast cancer risk of TBI and mantle radiotherapy. If this shows that the dose and risk are similar, then TBI patients are most likely to benefit from similar breast imaging follow up.
